# Electronic transport properties of the Al_0.5_TiZrPdCuNi alloy in the high-entropy alloy and metallic glass forms

**DOI:** 10.1038/s41598-022-06133-7

**Published:** 2022-02-10

**Authors:** Magdalena Wencka, Mitja Krnel, Andreja Jelen, Stanislav Vrtnik, Jože Luzar, Primož Koželj, Darja Gačnik, Anton Meden, Qiang Hu, Chaomin Wang, Sheng Guo, Janez Dolinšek

**Affiliations:** 1 Jožef Stefan Institute, Jamova 39, 1000 Ljubljana, Slovenia; 2 Institute of Molecular Physics, Polish Academy of Sciences, Smoluchowskiego 17, 60179 Poznan, Poland; 3 University of Ljubljana, Faculty of Mathematics and Physics, Jadranska 19, 1000 Ljubljana, Slovenia; 4 University of Ljubljana, Faculty of Chemistry and Chemical Technology, Večna pot 113, 1000 Ljubljana, Slovenia; 5 Institute of Applied Physics, Jiangxi Academy of Sciences, Changdong Road 7777, Nanchang, 330096 People’s Republic of China; 6 Industrial and Materials Science, Chalmers University of Technology, 41296 Göteborg, Sweden

**Keywords:** Materials science, Physics

## Abstract

High-entropy alloys (HEAs) are characterized by a simultaneous presence of a crystal lattice and an amorphous-type chemical (substitutional) disorder. In order to unravel the effect of crystal-glass duality on the electronic transport properties of HEAs, we performed a comparative study of the electronic transport coefficients of a 6-component alloy Al_0.5_TiZrPdCuNi that can be prepared either as a HEA or as a metallic glass (MG) at the same chemical composition. The HEA and the MG states of the Al_0.5_TiZrPdCuNi alloy both show large, negative-temperature-coefficient resistivity, positive thermopower, positive Hall coefficient and small thermal conductivity. The transport coefficients were reproduced analytically by the spectral conductivity model, using the Kubo-Greenwood formalism. For both modifications of the material (HEA and MG), contribution of phonons to the transport coefficients was found small, so that their temperature dependence originates predominantly from the temperature dependence of the Fermi–Dirac function and the variation of the spectral conductivity and the related electronic density of states with energy within the Fermi-level region. The very similar electronic transport coefficients of the HEA and the MG states point towards essential role of the immense chemical disorder.

## Introduction

High-entropy alloys (HEAs) are multi-component ($N\ge$ 5) crystalline solid solutions including random solutions and partially ordered ones^1,2^. HEAs possess enormous chemical (substitutional) disorder, similar to the one encountered in metallic glasses (MGs)^3^. Due to the simultaneous presence of a crystal lattice and an amorphous-type chemical disorder, HEA structures exhibit crystal-glass duality and can be conveniently termed as a “*metallic glass on a crystal lattice*”. Formation, stability, micro- and nano-structure and mechanical properties of HEAs have been widely investigated in the past^4–6^, while physical properties were less investigated, with the exception of magnetism and superconductivity^7–17^. A scarcely investigated topic of the HEAs is the electronic transport properties (electrical conductivity, thermoelectric power, electronic thermal conductivity and Hall coefficient), in relation to the crystal-glass duality. Crystallinity of the HEA structure introduces crystal-specific features like the electronic band structure with energy gaps between the bands, multi-branch Fermi surface and phononic dispersion relation, which largely determine the transport properties of crystalline solids. In MGs, there is no lattice and these features are absent. However, chemical disorder that is present to a similar extent in both the HEAs and the MGs also importantly influences the electronic transport properties. In this work, we address the role of the crystal-glass duality on the transport properties of HEAs by performing a comparative study of the transport coefficients of a 6-component metallic alloy Al_0.5_TiZrPdCuNi that can be prepared either as a HEA or as a MG at the same chemical composition. This has allowed us to unravel the effects of crystallinity and chemical disorder, in order to show how similar or different HEAs are from MGs.

There exists a rare class of near-equiatomic multicomponent alloys that can be prepared either in the crystalline state or as a bulk metallic glass (having the smallest dimension larger than 1 mm) at the same chemical composition. Such alloys are denoted as high-entropy bulk metallic glasses (HE-BMGs)^18–21^. The crystalline state of these alloys cannot be claimed to be a HEA, because the primary phase to form is always an intermetallic compound, rather than a solid solution phase. Upon crystallization, either during cooling or heating, an amorphous/intermetallic-compound composite is formed first, converting finally to fully intermetallic compounds (several of them) or perhaps a mixture of intermetallic compounds and solid solution phases. The Al_0.5_TiZrPdCuNi alloy can be also prepared in the crystalline or amorphous states at the same chemical composition^22,23^, but is rather unique in this class of alloys for two reasons. Firstly, the amorphous state can be prepared as ribbons of some 10-µm thickness by fast cooling of the spinning melt, which are too thin to be classified as a BMG, but conform to a MG. Secondly, the crystalline state of this alloy can indeed be prepared as a single-phase solid solution (HEA) with a bcc structure, either as melt-spun ribbons at a slower cooling rate or in the bulk rod form of 1.5 mm diameter by conventional Cu-mold casting. According to the available literature^18–23^, the Al_0.5_TiZrPdCuNi is the only composition that can form both the HEA and the MG states.

The single-phase bcc HEA state of the Al_0.5_TiZrPdCuNi is a quenched metastable state, because the equilibrium state of this system is a multi-phase crystalline state, composed of various intermetallic compounds. The multi-phase crystalline state as the equilibrium state can be expected on the basis of binary mixing enthalpies of the constituent elements (see Supplementary Table S1), which are highly negative (causing attraction) for most elemental pairs, with the most drastic examples of Pd-Zr ($\Delta {H}_{mix}^{PdZr}=$ − 91 kJ mol^–1^) and Pd-Ti ($\Delta {H}_{mix}^{PdTi}=$ – 65 kJ mol^–1^)^24,25^. The corresponding values of the total mixing enthalpy of this alloy $\Delta {H}_{mix}=$ – 46.7 kJ mol^–1^ and the atomic-size-difference (geometric) parameter $\delta =$ 8.8% are distinctly outside the ranges of crystalline solid solutions in the $\Delta {H}_{mix}$ vs. $\delta$ phase diagram^22,23,26^, indicating that the HEA state in the Al_0.5_TiZrPdCuNi alloy is of an unconventional type.

## Results

### Samples preparation and characterization

**Figure 1:**
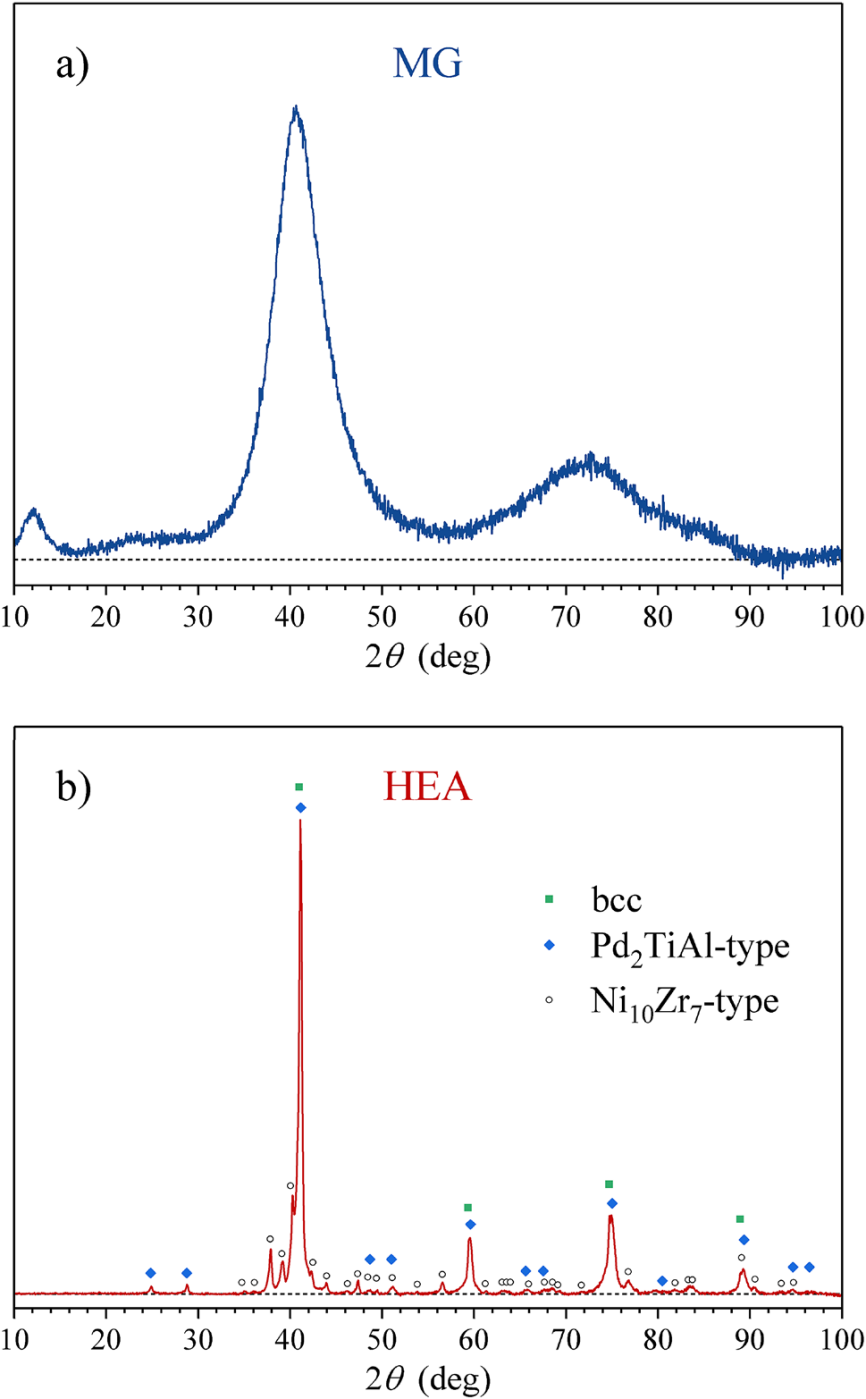
XRD patterns of the Al_0.5_TiZrPdCuNi alloy in (**a**) the MG state and (**b**) the HEA state.

**Figure 2:**
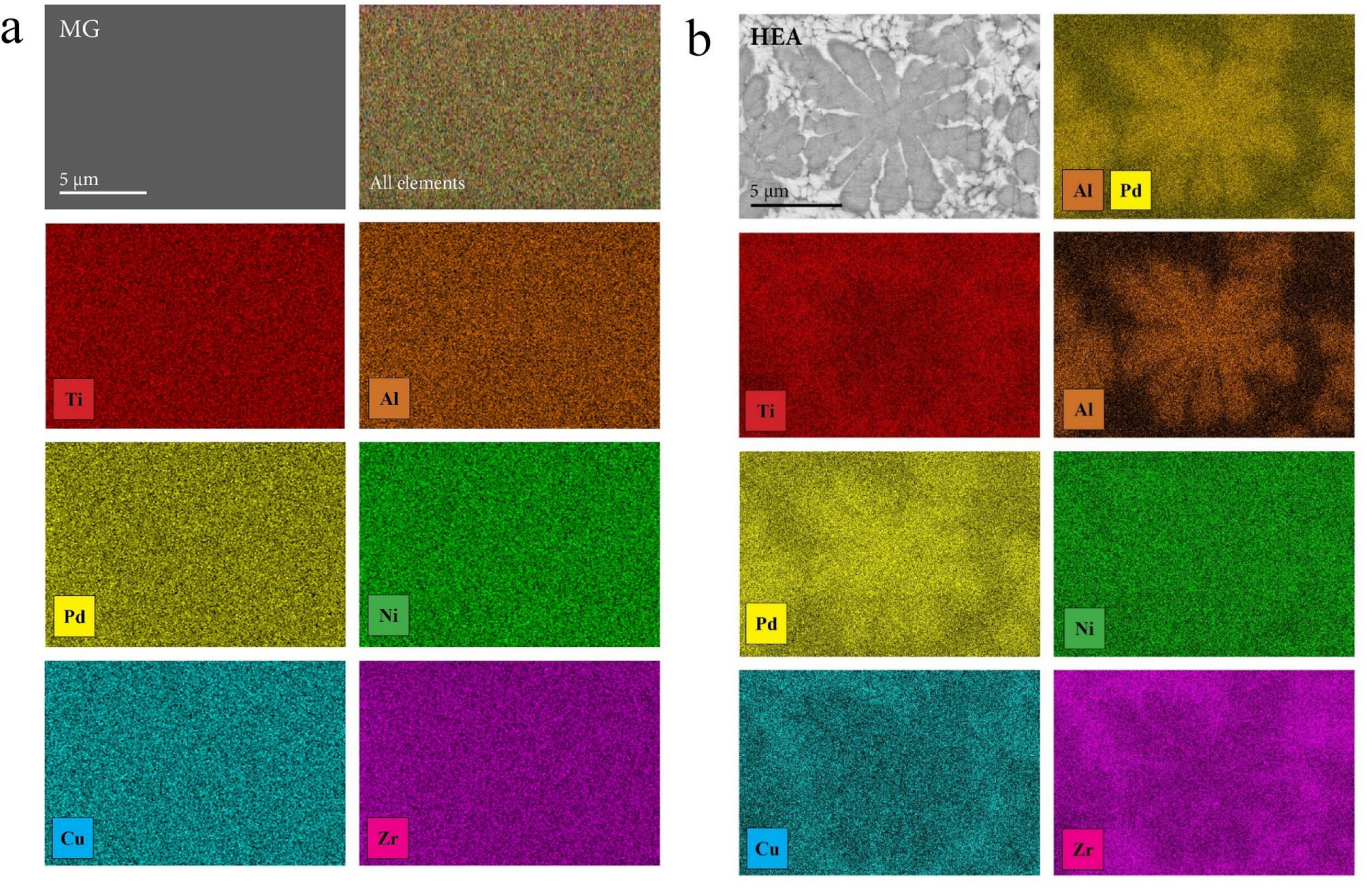
SEM EDS elemental maps of the Al_0.5_TiZrPdCuNi alloy in (**a**) the MG state and (**b**) the HEA state. The upper left panel shows the SEM BSE image.

The HEA and the MG samples of the alloy with the nominal composition Al_0.5_TiZrPdCuNi were prepared along the steps described in Refs.^22,23^. The MG sample was a ribbon of 20 µm thickness and 1 mm width. Its XRD pattern (Fig. 1a) is typical of amorphous structures, showing a broad halo at $2\theta \approx$ 40°. The SEM EDS elemental maps (Fig. 2a) indicate a homogeneous distribution of the six constituting elements on the µm scale. The EDS-determined composition (in at.%) is Al_7.0_Ti_21.1_Zr_15.3_Pd_14.0_Cu_21.3_Ni_21.3_. The HEA sample was a rod of 1.3 mm^2^ cross section. Its XRD pattern (Fig. 1b) reveals the presence of two major phases and one minor phase. The two major phases are (1) a bcc with the unit cell parameter $a=$ 3.10 Å and (2) a cubic, type Pd_2_TiAl (Heusler alloy), space group *Fm* $\overline{3 }$ *m*, $a=$ 6.20 Å (this unit cell edge is exactly twice that of the bcc phase, so that some XRD peaks of the two phases overlap), whereas the minor phase is orthorhombic, type Ni_10_Zr_7_, space group *Aea*2 (No. 41), $a=$ 9.20, $b=$ 9.20 and $c=$ 12.3 Å. The SEM BSE image and the elemental maps are shown in Fig. 2b, where the two major phases (one dark and one bright) are clearly discerned, each composed of crystallites of µm dimensions. Some small black inclusions are also visible at the borders between the two phases. High solid solubility of the elements is evident from the elemental maps. Al is mostly concentrated in the dark phase, which contains also all other elements, with a slight enhancement of Pd. Its EDS composition is Al_13.9_Ti_16.4_Zr_16.4_Pd_21.3_Cu_15.0_Ni_17.0_. The dark phase corresponds to the Pd_2_TiAl-type cubic phase, which is quite far from the ideal, stoichiometric composition due to the high solubility of the elements. The bright phase is a solid solution of all elements except Al (a small amount of Al is still dispersed in this phase) and corresponds to the bcc phase. Its EDS composition is Al_4.4_Ti_19.1_Zr_21.9_Pd_17.9_Cu_19.8_Ni_16.9_. The minor phase (orthorhombic, Ni_10_Zr_7_-type) cannot be easily identified in the SEM BSE image, very likely due to the high solid solubility of the elements. Neglecting the minor phase (of molar fraction less than 10%), the bcc (bright) phase occupies about 70% of the sample’s volume, whereas the Pd_2_TiAl-type cubic phase (dark) occupies about 30%.

The bcc and the Pd_2_TiAl-type phases are structurally related. The latter is also based on bcc, but with a specific atomic ordering that can be described in an enlarged, 2 $\times$ 2 $\times$ 2 unit cell (comprising eight bcc cells). For the Pd_2_TiAl stoichiometric composition, Ti and Al replace each other alternatively in the corners of the bcc cells, whereas Pd occupies the bcc cell centers (see Supplementary Fig. S1). Due to the high solubility of the elements, other elements also partially occupy the corners and the cell centers. In principle, the entire lattice of the Al_0.5_TiZrPdCuNi HEA phase (neglecting the minor phase) can be loosely viewed as a bcc structure with a unit cell parameter $a=$ 3.10 Å, where a larger part of it is chemically disordered and a smaller part is partially ordered (approximating rather well a HEA). This is somewhat different from the previous report on the Al_0.5_TiZrPdCuNi HEA phase^22^, which could be, under specific conditions, synthesized as a single-phase, chemically disordered bcc solution with a slightly larger lattice parameter $a=$ 3.20 Å (perhaps due to a slightly different chemical composition) in the entirety of its volume.

### Experimental transport coefficients, specific heat and magnetic properties

**Figure 3:**
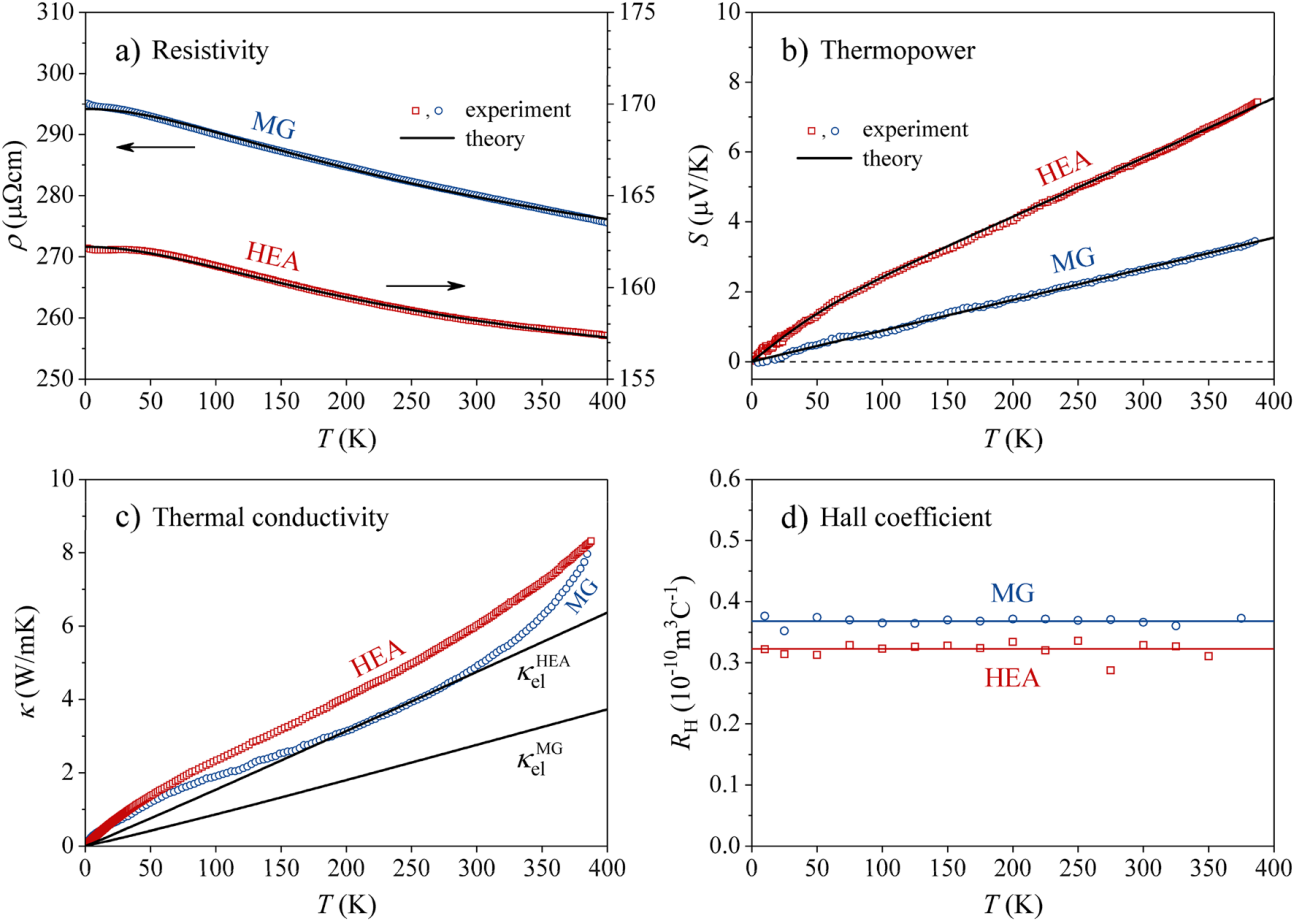
Transport coefficients of the Al_0.5_TiZrPdCuNi alloy in the HEA and MG states: (**a**) electrical resistivity, (**b**) thermopower, (**c**) thermal conductivity, and (**d**) Hall coefficient. Solid curves in the panels (**a**) and (**b**) are fits obtained with the spectral conductivity model, described in the text. In panel (**c**), the experimental data represent the total thermal conductivities ${\kappa }^{HEA}$ and ${\kappa }^{MG}$, whereas the solid curves are the calculated electronic thermal conductivities ${\kappa }_{el}^{HEA}$ and ${\kappa }_{el}^{MG}$.

**Figure 4:**
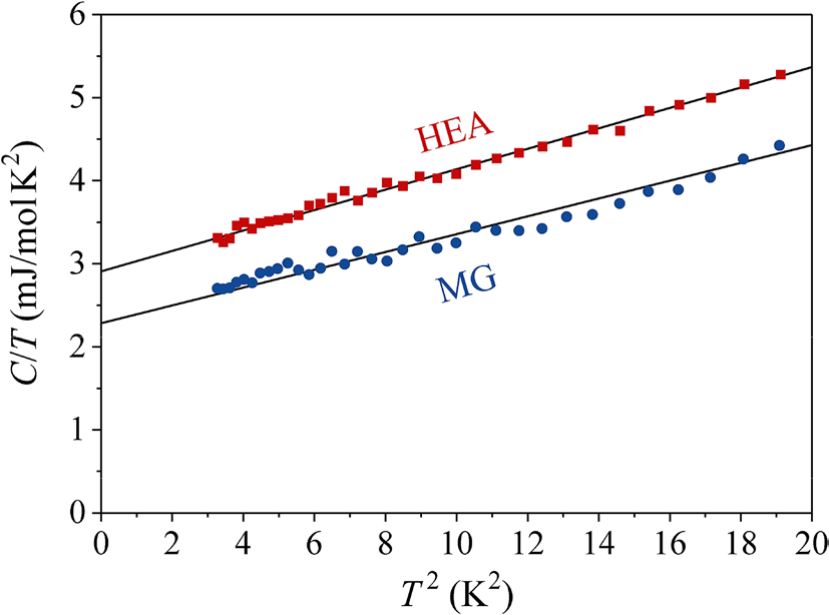
Low-temperature specific heat of the HEA and MG samples in a $C/T$ vs. ${T}^{2}$ plot. Solid lines are fits with the expression $C/T=\gamma +\alpha {T}^{2}$, and the fit parameters are given in the text.

**Figure 5:**
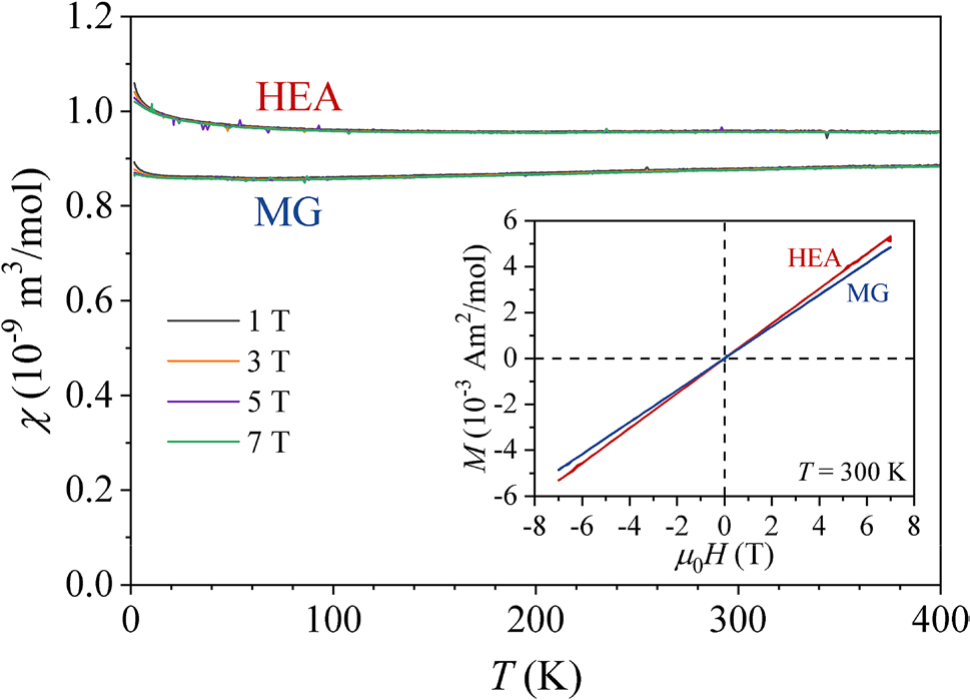
Magnetic susceptibility $\chi =M/H$ of the HEA and MG samples in magnetic fields 1, 3, 5 and 7 T (for each sample, the curves in different fields are indistinguishable on the graph, except in the $T\to$ 0 limit). The inset shows the isothermal magnetization curves, $M\left(H\right)$, at 300 K.

The transport coefficients, the specific heat and the magnetic properties were measured comparatively for the HEA and MG samples under identical experimental conditions, described in the “Methods” section. The electrical resistivity between 400 and 2 K is shown in Fig. 3a. Both samples exhibit negative-temperature-coefficient (NTC) resistivity that increases upon cooling, but there exists a pronounced difference in the magnitude of the resistivity and the NTC. For the MG sample, the 400-K resistivity amounts to ${\rho }_{400K}^{MG}=$ 277 µΩ cm, whereas the 2-K resistivity is ${\rho }_{2K}^{MG}=$ 295 µΩ cm, yielding the increase by ${NTC}^{MG}=\left({\rho }_{2K}^{MG}-{\rho }_{400K}^{MG}\right)/{\rho }_{400K}^{MG}=$ 7%. The resistivity of the HEA sample is considerably lower, with ${\rho }_{400K}^{HEA}=$ 157 µΩcm, ${\rho }_{2K}^{HEA}=$ 162 µΩcm and ${NTC}^{HEA}=$ 3%. The thermopower (the Seebeck coefficient $S$) of both samples is positive (Fig. 3b), increasing roughly linearly with the temperature (slight nonlinearity, more pronounced for the HEA sample, can be observed at temperatures below 100 K). The thermopower of the HEA sample is a factor of about two larger than that of the MG sample, amounting at 380 K to ${S}_{380K}^{HEA}=$ 7.3 µVK^–1^, as compared to ${S}_{380K}^{MG}=$ 3.2 µV K^–1^ of the MG sample. The thermal conductivity in the temperature range 1.8–390 K is shown in Fig. 3c, where it is seen that ${\kappa }^{HEA}$ of the HEA sample is a bit larger than ${\kappa }^{MG}$ of the MG sample in most of the investigated temperature range, but due to a somewhat faster increase of ${\kappa }^{MG}$ at higher temperatures, both samples reach the same 390-K value of ${\kappa }_{390K}^{MG}=$ ${\kappa }_{390K}^{HEA}=$ 8.2 W m^–1^ K^–1^. The Hall coefficient ${R}_{H}={E}_{y}/{j}_{x}{B}_{z}$ of both samples, measured in a magnetic field range ${\mu }_{0}H=$ ±9 T, is positive and temperature-independent (Fig. 3d), with the Hall coefficient of the MG sample, ${R}_{H}^{MG}=$ 3.7 $\times$ 10^–11^ m^3^ C^–1^ only insignificantly larger than that of the HEA sample, ${R}_{H}^{HEA}=$ 3.2 $\times$ 10^–11^ m^3^ C^–1^. The low-temperature specific heat $C$ between 1.8 and 4.5 K in a $C/T$ vs. ${T}^{2}$ plot is shown in Fig. 4, where it is observed that ${C}^{HEA}$ is a bit larger than ${C}^{MG}$. The fit with the standard expression $C/T=\gamma +\alpha {T}^{2}$, where $\gamma$ and $\alpha$ are the electronic and lattice specific heat coefficients, respectively, has yielded ${\gamma }^{HEA}=$ 2.91 mJ mol^–1^ K^–2^ and ${\gamma }^{MG}=$ 2.28 mJ mol^–1^ K^–2^. The Debye temperatures, calculated from $\alpha$ are ${\theta }_{D}^{HEA}=$ 251 ± 5 K and ${\theta }_{D}^{MG}=$ 262 ± 5 K. The electronic specific heat coefficient is directly proportional to the electronic DOS at the Fermi energy $g\left({\varepsilon }_{F}\right)$ via the relation $\gamma =\left({\pi }^{2}/3\right){k}_{B}^{2}g\left({\varepsilon }_{F}\right)$, which can also be written as $\gamma =$ 2.358 $g\left({\varepsilon }_{F}\right)$, where $\gamma$ is given in units [mJ mol^–1^ K^–2^] and $g\left({\varepsilon }_{F}\right)$ is then obtained in units [states/(eV $\cdot$ atom)]^27^. From this relation we obtain ${g}^{HEA}\left({\varepsilon }_{F}\right)=$ 1.23 states/(eV $\cdot$ atom) and ${g}^{MG}\left({\varepsilon }_{F}\right)=$ 0.97 states/(eV $\cdot$ atom), with their ratio ${g}^{HEA}\left({\varepsilon }_{F}\right)/{g}^{MG}\left({\varepsilon }_{F}\right)=$ 1.27. The field-cooled magnetic susceptibility $\chi =M/H$ of both samples, measured between 400 and 2 K in several magnetic fields between 1 and 7 T (Fig. 5) is positive, field-independent and also practically temperature independent (a small Curie upturn due to extrinsic magnetic impurities can be observed at low temperatures). The isothermal magnetization cures, $M\left(H\right)$, are shown in the inset of Fig. 5, exhibiting linear paramagnetic behavior up to the highest field of 7 T. These results demonstrate that Ni is in a non-magnetic state and both structural forms (HEA and MG) of the Al_0.5_TiZrPdCuNi alloy exhibit Pauli spin paramagnetism of the conduction electrons. The magnetic susceptibility is a sum of the Pauli paramagnetic susceptibility and the Larmor diamagnetic susceptibility of closed atomic shells, $\chi ={\chi }_{P}+{\chi }_{L}$ (the Landau orbital diamagnetic contribution can be neglected due to the enormous chemical and structural disorder in the samples), where ${\chi }_{P}$ and ${\chi }_{L}$ are of the same order of magnitude. An estimate of the Larmor susceptibility from literature tables^28^, using different ionization states of the elements yields the range ${\chi }_{L}=$ − [0.127, 0.153]$\times$ 10^–9^ m^3^ mol^–1^, which is to be contrasted with the total susceptibility values (e.g., at 300 K) ${\chi }^{HEA}=$ 0.98 $\times$ 10^–9^ m^3^ mol^–1^ and ${\chi }^{MG}=$ 0.89 $\times$ 10^–9^ m^3^ mol^–1^. The comparison shows that $\left|{\chi }_{L}\right|$ accounts for about 15% of the total susceptibility, wherefrom we can make a rough estimate of the Pauli susceptibility ratio of the HEA and MG samples as ${\chi }_{P}^{HEA}/{\chi }_{P}^{MG}\approx$ 1.1. The Pauli spin susceptibility has a simple relation to the electronic density of states at the Fermi energy, $g\left({\varepsilon }_{F}\right)$, via the relation ${\chi }_{P}={\mu }_{0}{\mu }_{B}^{2}g\left({\varepsilon }_{F}\right)$, where ${\mu }_{0}$ is the permeability of vacuum and ${\mu }_{B}$ is the Bohr magneton. The susceptibility analysis then yields the ratio ${g}^{HEA}\left({\varepsilon }_{F}\right)/{g}^{MG}\left({\varepsilon }_{F}\right)\approx$ 1.1, which is in fair agreement with the value 1.27 determined from the specific heat (where the latter should be considered as more precise).

### Theoretical analysis of the transport coefficients

The electronic transport coefficients (electrical conductivity $\sigma =1/\rho$, thermopower $S$, electronic thermal conductivity ${\kappa }_{el}$ and Hall coefficient ${R}_{H}$) were analyzed analytically by the spectral conductivity model, using Kubo–Greenwood formalism^29,30^ and the assumptions that (1) the HEA and MG structures are both spatially isotropic (for the HEA structure, this follows from the average cubic symmetry of the lattice), so that the tensorial transport coefficients reduce to scalars and (2) the effect of phonons is minor, so that the temperature dependence of the electronic transport coefficients originates predominantly from the temperature dependence of the Fermi–Dirac (FD) function. The second assumption follows from the fact that the immense chemical (substitutional) disorder and the associated topological disorder (lattice distortions) due to different atomic sizes in the HEA structure or complete absence of the lattice in the MG structure represent quenched defects, which break translational periodicity of the system. This causes elastic scattering of the conduction electrons at an extremely rapid rate at any temperature, providing the main scattering mechanism for the electronic transport phenomena.

Within the spectral conductivity model, the coefficients $\sigma \left(T\right)$, $S\left(T\right)$ and ${\kappa }_{el}\left(T\right)$ can all be derived from a single, material-dependent quantity, the spectral conductivity $\sigma \left(\varepsilon \right)$, which is related to the electronic DOS $g\left(\varepsilon \right)$ via the Einstein relation

1 $$\sigma \left(\varepsilon \right)=\left({e}^{2}/V\right)g\left(\varepsilon \right)D\left(\varepsilon \right),$$

where $D\left(\varepsilon \right)$ is the electronic spectral diffusivity and $V$ is the sample volume. We shall further use the approximation that the energy dependence of $D\left(\varepsilon \right)$ can be neglected in the vicinity of the Fermi level ${\varepsilon }_{F}$, by taking $D\left(\varepsilon \right)\approx D\left({\varepsilon }_{F}\right)$, so that the shape of $\sigma \left(\varepsilon \right)$ within the Fermi-level region is the same as the shape of the DOS $g\left(\varepsilon \right)$ (to a constant multiplicative factor).

Within the above model, the electrical conductivity $\sigma \left(T\right)$, the Seebeck coefficient $S\left(T\right)$ and the electronic thermal conductivity ${\kappa }_{el}\left(T\right)$ are calculated from

2 $$\sigma \left(T\right)=\int d\varepsilon \sigma \left(\varepsilon \right) \left(-\partial f/\partial \varepsilon \right),$$

3 $$S\left(T\right)={\left[eT\sigma \left(T\right)\right]}^{-1}\int d\varepsilon \sigma \left(\varepsilon \right) \left(\varepsilon -\mu \right) \left(-\partial f/\partial \varepsilon \right),$$

and

4a $${\kappa }_{el}\left(T\right)=\left[{L}_{22}\left(T\right)/{e}^{2}T\right]-T\sigma \left(T\right){S}^{2}\left(T\right),$$

with

4b $${L}_{22}\left(T\right)=\int d\varepsilon \sigma \left(\varepsilon \right) {\left(\varepsilon -\mu \right)}^{2}\left(-\partial f/\partial \varepsilon \right).$$

The parameter $e$ in the above equations is the electric charge of the carriers (its sign distinguishes between electrons and holes), $f={\left\{exp\left[\left(\varepsilon -\mu \right)/{k}_{B}T\right]+1\right\}}^{-1}$ is the FD function and $\mu$ is the chemical potential. At low temperatures (such as room temperature), $\mu$ can be expressed as^31^

5 $$\mu \left(T\right)\approx {\varepsilon }_{F}-{\left({k}_{B}T\right)}^{2}\left({\pi }^{2}/6\right){\left[dln g\left(\varepsilon \right)/d\varepsilon \right]}_{{\varepsilon }_{F}}={\varepsilon }_{F}-\xi {T}^{2}.$$

Within the $D\left(\varepsilon \right)\approx D\left({\varepsilon }_{F}\right)$ approximation, we can replace $g\left(\varepsilon \right)$ in Eq. (5) by $\sigma \left(\varepsilon \right)$, which allows to relate the parameter $\xi$ to the Seebeck coefficient using Mott’s formula

6a $${S}^{Mott}\left(T\right)=\left({\pi }^{2}/3\right)\left({k}_{B}^{2}/e\right){\left[dln \sigma \left(\varepsilon \right)/d\varepsilon \right]}_{{\varepsilon }_{F}}T$$

as

6b $$\xi =\left(e/2\right)\left[{S}^{Mott}\left(T\right)/T\right].$$

The experimental thermopower $S\left(T\right)$ data can hence be used to set the starting value of the $\xi$ fit parameter. In practical terms, the theoretical analysis of the transport coefficients is done by assuming a specific functional form of the spectral conductivity $\sigma \left(\varepsilon \right)$ and then performing simultaneous fitting of the quantities $\sigma \left(T\right)$ and $S\left(T\right)$. After satisfactory fits are obtained, ${\kappa }_{el}\left(T\right)$ is calculated theoretically, because this quantity is not available experimentally, but only the total thermal conductivity $\kappa \left(T\right)$ (that includes the phononic contribution) is measured. A comparison of the theoretical ${\kappa }_{el}\left(T\right)$ and the experimental $\kappa \left(T\right)$ is then used to estimate the residual phononic thermal conductivity of the HEA and MG phases.

Regarding the Hall coefficient, the magneto-transport is less understood. A positive Hall coefficient, ${R}_{H}>0$, has been reported for many amorphous transition metals and amorphous alloys containing transition metals^32–34^, which made the sign of ${R}_{H}$ in disordered materials a well-known problem^35–37^. Within the Kubo formalism, the Hall coefficient in the weak-field limit is given by

7 $${R}_{H}=\left(1/B\right){\sigma }_{xy}\left(B\right)/{\sigma }_{xx}^{2},$$

where the magnetic field $B$ points along the $z$ direction, whereas the external electric field and the Hall field point along the $x$ and the negative $y$ direction, respectively. ${\sigma }_{xy}$ denotes the off-diagonal element of the conductivity tensor and ${\sigma }_{xx}$ is the diagonal element. Their analytical expressions for amorphous structures can be found e.g. in Ref.^38^. Both tensor elements are proportional to the square of the electric charge, ${\sigma }_{xx}, {\sigma }_{xy}$ $\propto {e}^{2}$, and hence do not distinguish between electrons and holes, i.e. the sign of the charge carriers does not determine directly the sign of ${R}_{H}$. It was then conjectured^39^ that the sign of the Hall coefficient is correlated with the negative sign of the derivative of the electronic DOS at the Fermi energy, ${R}_{H}\propto -{\left(dg/d\varepsilon \right)}_{{\varepsilon }_{F}}$. This consideration was supported by other literature reports^34,37,38^, including a numerical re-examination of the problem^40^. In our analysis of the Hall coefficient of the HEA and MG samples, we shall check the validity of the ${R}_{H}\propto -{\left(dg/d\varepsilon \right)}_{{\varepsilon }_{F}}$ relation, i.e. whether the sign of the Hall coefficient equals the sign of the negative DOS derivative within the Fermi level region. An independent check here is that the sign of ${R}_{H}$ should be in agreement with the sign of the thermopower $S$, which also depends on the DOS derivative at ${\varepsilon }_{F}$, in a product with the sign of the charge carriers $e$ (as can be seen from Eq. (6a)).

In a measurement of the electronic transport coefficients, the experimentally observable part of the spectral conductivity $\sigma \left(\varepsilon \right)$ is determined by the position and width of the symmetric, bell-shaped FD function derivative, $-df/d\varepsilon$, which is centered at the chemical potential $\mu$. Its full width at half maximum (FWHM) ${\Delta }_{f}=$ 3.5 ${k}_{B}T$ amounts at room temperature to ${\Delta }_{f}^{300K}=$ 90 meV, whereas at $T=$ 2 K it narrows to ${\Delta }_{f}^{2K}=$ 0.6 meV. In the $T\to 0$ limit, $-df/d\varepsilon$ converges to a delta function $\delta \left(\varepsilon -{\varepsilon }_{F}\right)$ and Eq. (2) yields the relation between the zero-temperature residual resistivity $\rho \left(0\right)$ and the spectral conductivity at ${\varepsilon }_{F}$ as $\rho \left(0\right)=1/\sigma \left({\varepsilon }_{F}\right)$. Due to the temperature dependence of $\mu$ according to Eq. (5), the experimentally observable part of $\sigma \left(\varepsilon \right)$ is shifting with temperature on the energy axis, so that different parts of $\sigma \left(\varepsilon \right)$ and the DOS $g\left(\varepsilon \right)$ contribute to the integrals in Eqs. (2), (3), (4b) and (7). An estimate of $\xi$ from the thermopower data shown in Fig. 3b via Eq. (6b) gives for the HEA sample ${\xi }^{HEA}=$ –9.6 $\times$ 10^–9^ eVK^–2^, yielding at 380 K a tiny shift of the chemical potential from ${\varepsilon }_{F}$ by ${\mu }_{380K}^{HEA}-{\varepsilon }_{F}\approx$ 1.4 meV. An estimate of $\xi$ for the MG sample gives ${\xi }^{MG}=$ –4.2 $\times$ 10^–9^ eVK^–2^ and ${\mu }_{380K}^{MG}-{\varepsilon }_{F}\approx$ 0.6 meV. The shift of $\mu$ on the energy scale is very small, so that the electronic transport coefficients are sensitive to a narrow portion of $\sigma \left(\varepsilon \right)$ (and the DOS $g\left(\varepsilon \right)$) in the energy interval of about ± 100 meV around ${\varepsilon }_{F}$. Significant temperature dependence of the transport coefficients can be expected when the DOS changes with energy substantially on this scale.

Modeling the spectral conductivity $\sigma \left(\varepsilon \right)$ can be performed via different trial functions, a common property of which should be a substantial variation within the experimentally observable energy range. We shall use the model by Landauro and Solbrig, originally developed for quasicrystals^41–45^. A common feature that quasicrystals share with the HEAs and MGs is the low contribution of phonons to the transport coefficients, because phonon propagation in a non-periodic quasicrystalline structure is strongly hindered. Within this model, the spectral resistivity (the inverse spectral conductivity) $\rho \left(\varepsilon \right)=1/\sigma \left(\varepsilon \right)$ is constructed as a superposition of two Lorentzians (the details are given in the Supplementary Information). Choosing proper positions and widths of the Lorentzians on the energy axis within the Fermi-level region allows constructing $\sigma \left(\varepsilon \right)$ that exhibits either simple monotonous, positive- or negative-slope behavior, or a minimum (like the pseudogap in the DOS at ${\varepsilon }_{F}$ in quasicrystals) or a maximum, with possible fine structure on the scale of a few meV. The actual shape of $\sigma \left(\varepsilon \right)$ is then adjusted by a simultaneously fitting of the $\rho \left(T\right)$ and $S\left(T\right)$ experimental data.

**Figure 6:**
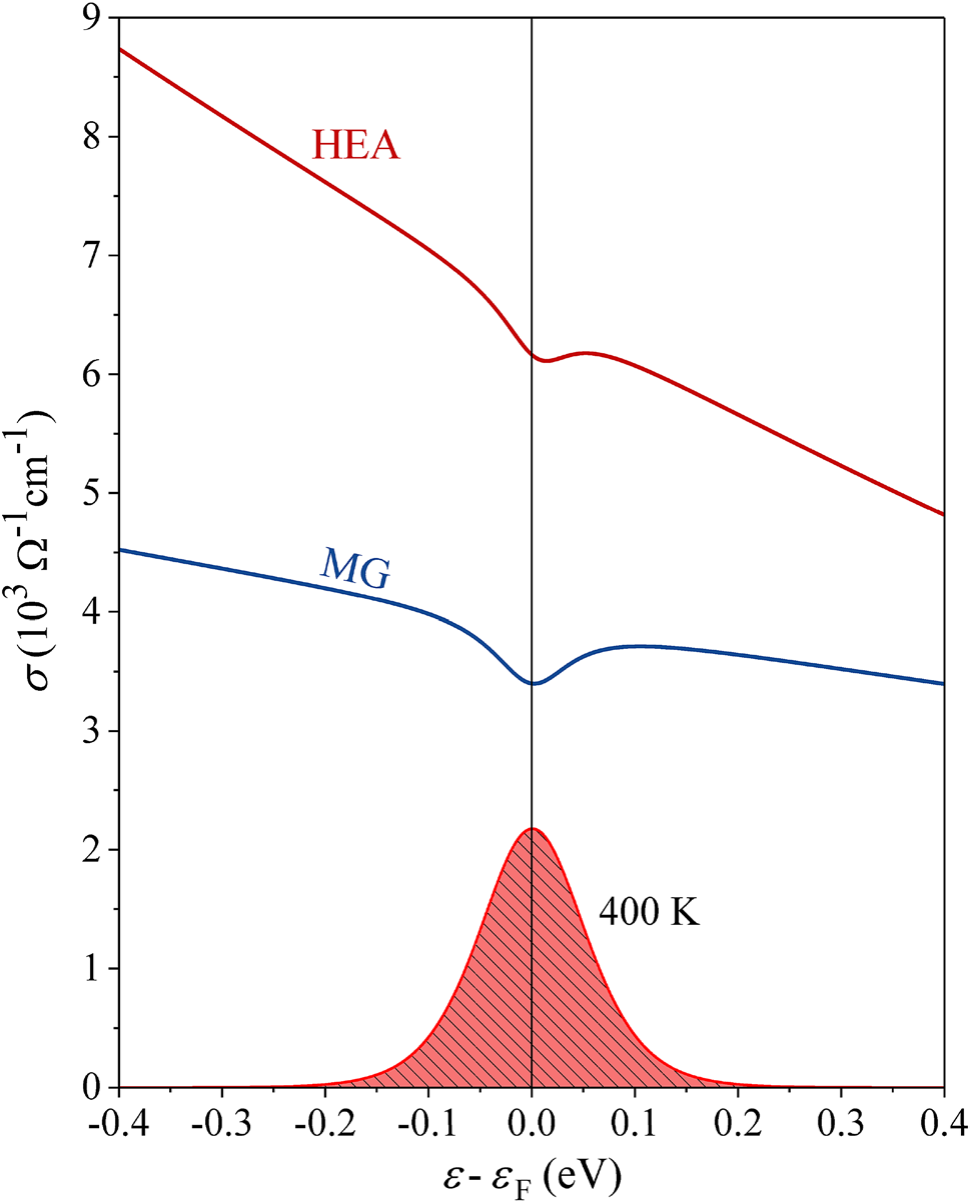
Spectral conductivities ${\sigma }^{HEA}\left(\varepsilon \right)$ and ${\sigma }^{MG}\left(\varepsilon \right)$, determined from simultaneous fitting of the electrical resistivity $\rho \left(T\right)$ and the thermopower $S\left(T\right)$ (the sets of fit parameters are given in Supplementary Table S2). The bell-shaped derivative of the FD function, $-\partial f/\partial \varepsilon$, at 400 K is shown at the bottom of the graph (its vertical scale does not conform to the $\sigma \left(\varepsilon \right)$ scale).

The fits of $\sigma \left(T\right)$ and $S\left(T\right)$ of the HEA and MG samples with Eqs. (2) and (3) are shown by solid curves in Fig. 3a,b. Excellent fits were obtained by the spectral conductivity functions presented in Fig. 6 (the sets of fit parameters determining ${\sigma }^{HEA}\left(\varepsilon \right)$ and ${\sigma }^{MG}\left(\varepsilon \right)$ are given in Supplementary Table S2). For converging results, the integrations had to be performed over an energy interval of ± 10 ${k}_{B}T$ around ${\varepsilon }_{F}$, which amounts to ± 0.17 eV at 400 K. This value defines the experimentally observed portions of the $\sigma \left(\varepsilon \right)$ and $g\left(\varepsilon \right)$ quantities. For both the HEA and the MG samples, the spectral conductivity is a negative-sloping function within the Fermi-level region, with a small dip at ${\varepsilon }_{F}$. The ${\sigma }^{HEA}\left({\varepsilon }_{F}\right)$ is by a factor of 1.8 larger than ${\sigma }^{MG}\left({\varepsilon }_{F}\right)$, accounting for the fact that ${\rho }^{HEA}\left(0\right)$ is smaller than ${\rho }^{MG}\left(0\right)$ by the same factor. The final values of the fit parameters that shift the chemical potential on the temperature axis are ${\xi }^{HEA}=$ –15.0 $\times$ 10^–9^ eVK^–2^ and ${\xi }^{MG}=$ –4.6 $\times$ 10^–9^ eVK^–2^, very close to the starting values determined from the Mott formula of Eq. (6b). In Fig. 6, the function $-\partial f/\partial \varepsilon$ is also shown at the temperature of 400 K, where the shift of the maximum relative to ${\varepsilon }_{F}$ due to the temperature-dependent chemical potential is unobservable on this energy scale.

The negative-sloping $\sigma \left(\varepsilon \right)$ within the Fermi-level region and the assumption of negatively charged carriers, $e=-\left|e\right|$ (electrons) reproduce quantitatively the NTC electrical resistivity and the positive thermopower with all their nonlinear features in the entire measured temperature range 2–400 K for both samples. Knowing $\sigma \left(T\right)$ and $S\left(T\right)$, the electronic thermal conductivity ${\kappa }_{el}\left(T\right)$ was calculated from Eqs. (4a,b) and is presented by solid lines in Fig. 3c. It is observed that the calculated ${\kappa }_{el}^{HEA}\left(T\right)$ and ${\kappa }_{el}^{MG}\left(T\right)$ account for the majority part of the experimental total thermal conductivities ${\kappa }^{HEA}\left(T\right)$ and ${\kappa }^{MG}\left(T\right)$, supporting the assumption that the phononic thermal conductivity ${\kappa }_{ph}$ is small for both samples. The negative derivative $d\sigma /d\varepsilon <0$ and the associated $dg/d\varepsilon <0$ within the Fermi-level region also consistently explain the positive sign of the Hall coefficient ${R}_{H}$ and hence support the relation ${R}_{H}\propto -{\left(dg/d\varepsilon \right)}_{{\varepsilon }_{F}}$ for the investigated chemically and topologically disordered HEA and MG samples.

## Discussion and conclusions

The electronic transport coefficients of the HEA and MG states of the Al_0.5_TiZrPdCuNi alloy reveal certain similarities as well as differences. The two states have in common the enormous chemical disorder, but differ in the presence/absence of a crystal lattice. In both cases, the contribution of phonons to the transport coefficients is small. While phonons cannot propagate in the amorphous state due to the absence of a crystal lattice, the lattice in the HEA state is unsuitable for the phonon propagation because of the topological distortion and random distribution of masses (different chemical elements) on the lattice sites. An exception are long-wavelength acoustic phonons, which “see” both structures as an elastic continuum and still contribute to the transport phenomena. Due to the weak phonon contribution, the temperature dependence of the electronic transport coefficients originates predominantly from the temperature dependence of the FD function and the variation of the spectral conductivity $\sigma \left(\varepsilon \right)$ and the related electronic DOS $g\left(\varepsilon \right)$ with energy within the Fermi-level region. For the Al_0.5_TiZrPdCuNi alloy, ${\sigma }^{HEA}\left(\varepsilon \right)$ and ${\sigma }^{MG}\left(\varepsilon \right)$ are both decreasing functions in that energy range, with some fine structure on the 10-meV scale very close to ${\varepsilon }_{F}$. Under the realistic assumption that the spectral diffusivity $D\left(\varepsilon \right)$ does not change significantly within the Fermi-level region, the electronic DOSs ${g}^{HEA}\left(\varepsilon \right)$ and ${g}^{MG}\left(\varepsilon \right)$ show the same shape as their respective spectral conductivities. Knowing the DOS at ${\varepsilon }_{F}$ values from the specific heat measurements (recall that their ratio is ${g}^{HEA}\left({\varepsilon }_{F}\right)/{g}^{MG}\left({\varepsilon }_{F}\right)=$ 1.27), while the $T\to$ 0 resistivities yield ${\sigma }^{HEA}\left({\varepsilon }_{F}\right)/{\sigma }^{MG}\left({\varepsilon }_{F}\right)={\rho }^{MG}\left(0\right)/{\rho }^{HEA}\left(0\right)=$ 1.8, the Einstein relation of Eq. (1) yields the ratio of the spectral diffusivities ${D}^{HEA}\left({\varepsilon }_{F}\right)/{D}^{MG}\left({\varepsilon }_{F}\right)=$ 1.4. By assuming that the electronic diffusion constant can be described by the Einstein formula $D={\mu }_{e}{k}_{B}T/e$, valid for an electrically charged Brownian particle of charge $e$ in an electric field $E$ with mobility ${\mu }_{e}$ (defined as the ratio of the electronic drift velocity ${v}_{d}$ to the magnitude of the electric field, ${\mu }_{e}={v}_{d}/E$), we can estimate the ratio of the electronic mobilities in the HEA and MG phases to be ${\mu }_{e}^{HEA}/{\mu }_{e}^{MG}\approx$ 1.4, i.e. the mobilities are about the same. This number is independently confirmed from the Hall coefficient and the conductivity using expression ${\mu }_{e}=\sigma {R}_{H}$, which yields a very similar value ${\mu }_{e}^{HEA}/{\mu }_{e}^{MG}=\left({\sigma }^{HEA}\left({\varepsilon }_{F}\right)/{\sigma }^{MG}\left({\varepsilon }_{F}\right)\right)\cdot \left({R}_{H}^{HEA}/{R}_{H}^{MG}\right)\approx$ 1.5, pointing toward the essential role of chemical disorder in determining the electronic transport coefficients of both the HEA and the MG states, whereas the presence/absence of the (topologically distorted) crystal lattice is of minor importance, but still experimentally observable. The enormous chemical and topological disorders represent quenched defects in the structure, which scatter the electrons elastically at an extremely rapid rate at any temperature and strongly reduce the transport phenomena relative to the chemically ordered crystals. The order of magnitude of the relaxation time ${\tau }_{0}$ (the mean time between two elastic scattering events) can be estimated from the Einstein conductivity of Eq. (1), by considering that the diffusion constant is given by $D={l}_{0}^{2}/{\tau }_{0}$, where ${l}_{0}$ is the mean free path. Due to the immense disorder, it is reasonable to consider that the mean free path in the HEA lattice assumes its limiting (constant) value ${l}_{0}\approx a$, where $a=$ 3.1 Å is the unit cell length. For the MG sample, ${l}_{0}$ can be taken as the nearest-neighbor distance, again about 3 Å. Taking the experimental $g\left({\varepsilon }_{F}\right)$ and ${\rho }_{2K}$ values, we obtain ${\tau }_{0}\sim$ 10^–15^ s for both structural forms of the material. This is to be contrasted with pure metals near absolute zero, where ${\tau }_{0}\sim$ 10^–9^ s is typical and ${l}_{0}$ is on the order of 10^6^ atomic distances (becoming ${\tau }_{0}\sim$ 10^–14^ s and ${l}_{0}$ about 100 atomic distances at room temperature). From the transport-properties point of view, HEAs appear very similar to MGs, but are quite different from translationally periodic, chemically ordered crystals.

## Methods

XRD patterns were recorded on a PANalytical X'Pert PRO MPD X-ray powder diffractometer using Cu Kα_1_ radiation ($\lambda =$ 1.54056 Å). SEM BSE imaging and EDS measurements of the chemical composition and elemental mapping were conducted on a focused ion beam scanning electron microscope FEI HeliosNanolab 650, equipped with EDS system from Oxford Instruments with X-max SDD detector. Electrical resistivity, thermoelectric power, thermal conductivity, Hall coefficient and specific heat were measured by a Quantum Design Physical Property Measurement System (PPMS 9 T). Magnetic properties were recorded on a Quantum Design MPMS3 SQUID magnetometer.

## Supplementary Information

Supplementary Information.

## Data Availability

The datasets generated during and/or analyzed during the current study are available from the corresponding author on reasonable request.
