# Supplementary material for: Electronic transport properties of the Al0.5TiZrPdCuNi alloy in the high-entropy alloy and metallic glass forms
Source: Sci Rep. 2022 Feb 10;12:2271. doi: 10.1038/s41598-022-06133-7 (PMC8831620; doi:10.1038/s41598-022-06133-7)
Supplement: Supplementary file 1 — Supplementary Information. [file 41598_2022_6133_MOESM1_ESM.pdf]

Supplementary Information for

Electronic transport properties of the $Al_{0.5}$TiZrPdCuNi alloy in the high-entropy alloy and metallic glass forms

Magdalena Wencka[1,2], Mitja Krnel[1], Andreja Jelen[1], Stanislav Vrtnik[1], Jože Luzar[1], Primož Koželj[1,3], Darja Gačnik[1], Anton Meden[4], Qiang Hu[5], Chaomin Wang[5], Sheng Guo[6], Janez Dolinšek[1,3]

[1] *Jožef Stefan Institute, Jamova 39, SI-1000 Ljubljana, Slovenia*

[2] *Institute of Molecular Physics, Polish Academy of Sciences, Smoluchowskiego 17, PL-60-179 Poznań, Poland*

[3] *University of Ljubljana, Faculty of Mathematics and Physics, Jadranska 19, SI-1000 Ljubljana, Slovenia*

[4] *University of Ljubljana, Faculty of Chemistry and Chemical Technology, Večna pot 113, SI-1000 Ljubljana, Slovenia*

[5] *Institute of Applied Physics, Jiangxi Academy of Sciences, Changdong Road 7777, Nanchang 330096, PR China*

[6] *Industrial and Materials Science, Chalmers University of Technology, SE-41296 Göteborg, Sweden*

Supplementary Table S1. Binary mixing enthalpies (in $kJmol^{-1}$) for unlike atom pairs constituting the $Al_{0.5}TiZrPdCuNi$ alloy (adapted from refs.[24,25] of the main paper).

Al	–30	–44	–46	–1	–22
–30	Ti	0	–65	–9	–35
–44	0	Zr	–91	–23	–49
–46	–65	–91	Pd	–14	0
–1	–9	–23	–14	Cu	4
–22	–35	–49	0	4	Ni

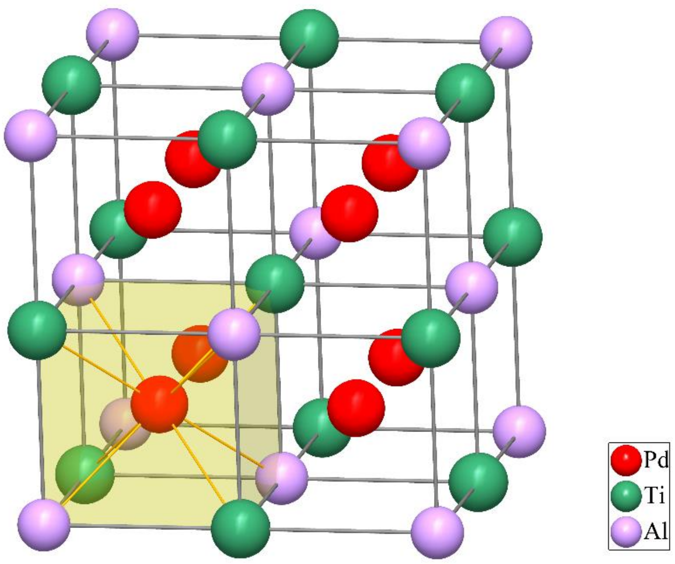

Supplementary Figure S1. Unit cell of the stoichiometric Pd_2TiAl cubic phase (Heusler alloy, space group $Fm\bar{3}m$, $a = 6.20$ Å).

Spectral conductivity function of the Landauro-Solbrig model. Within the Landauro-Solbrig model (refs.[41–43] of the main paper), the spectral resistivity (the inverse spectral conductivity) $\rho(\varepsilon) = 1/\sigma(\varepsilon)$ is constructed as a superposition of two Lorentzians

$$\rho(\varepsilon) = A\left\{\left[\frac{1}{\pi}\frac{\gamma_1}{(\varepsilon-\delta_1)^2+\gamma_1^2}\right] + \beta\left[\frac{1}{\pi}\frac{\gamma_2}{(\varepsilon-\delta_2)^2+\gamma_2^2}\right]\right\}, \quad \text{(S1)}$$

where each Lorentzian is characterized by height $1/\pi\gamma_i$, full width at half maximum (FWHM) of $2\gamma_i$ and position δ_i relative to the Fermi energy (taken as $\varepsilon_F = 0$), whereas β is the relative weight of the Lorentzians. Simultaneous fit of the $\rho(T)$ and $S(T)$ quantities with equations (2) and (3) then yields the set of parameters (A, β, δ_i and γ_i) that determine the shape of the spectral conductivity $\sigma(\varepsilon)$ within the experimentally observable Fermi-level region. The sets of parameters obtained for the HEA and BMG samples that yield the spectral conductivities shown in Fig. 6 are collected in Supplementary Table S2.

Supplementary Table S2: Parameters of the spectral resistivity function $\rho(\varepsilon)$ given by equation (S1) for the HEA and BMG samples, obtained from the simultaneous fits of the $\rho(T)$ and $S(T)$ data.

sample	A (μΩcm eV)	δ_1 (meV)	γ_1 (meV)	β	δ_2 (eV)	γ_2 (eV)
HEA	1.38	6.1	38.9	2481	2.37	0.88
BMG	5.49	0.43	43.3	1640	4.48	2.21
